# Uniaxial Compression Failure Behavior and Energy Evolution of Sandstone–Marble Waste Powder Concrete Composites

**DOI:** 10.3390/s26134219

**Published:** 2026-07-03

**Authors:** Xiang Huang, Jiahao Cao, Shuguang Zhang, Jiaming Li, Zongyuan Pan, Shibin Tang

**Affiliations:** 1 Guangxi Key Laboratory of Geomechanics and Geotechnical Engineering, Guilin University of Technology, Guilin 541004, China; huangxiang@glut.edu.cn (X.H.); 1020250888@glut.edu.cn (J.C.); zhangsg@glut.edu.cn (S.Z.); 2 Institute of Karst Geology, Chinese Academy of Geological Sciences (CAGS), Guilin 541004, China; panzongyuan@mail.cgs.gov.cn; 3 School of Civil Engineering, Dalian University of Technology, Dalian 116024, China; tang_shibin@dlut.edu.cn

**Keywords:** sandstone–marble waste powder concrete composite, joint roughness coefficient, uniaxial compression, energy evolution, acoustic emission, digital image correlation

## Abstract

Sandstone–marble waste powder concrete composite structures serve as common load-bearing systems in tunnels, underground caverns, and similar engineering projects, where the interface roughness characteristics directly govern their overall stability and service safety. To investigate the influence of interface roughness on the failure behavior of the composite, four groups of sandstone–concrete composite specimens made with marble waste powder concrete were prefabricated with different joint roughness coefficients (*JRC* = 0, 7.84, 17.99, 20.79). The concrete matrix was prepared with marble waste powder incorporated at 25 wt% of the total binder, corresponding to 20.45 wt% of the total mixture, and the water-to-binder ratio was 0.20. Uniaxial compression tests were conducted with synchronous acoustic emission (AE) and digital image correlation (DIC) monitoring to examine the roughness-dependent mechanical response, energy evolution, damage activity, and strain localization of the composites. The results show that the peak stress and elastic modulus of the composite increase continuously with increasing *JRC*. When *JRC* increases from 0 to 20.79, the peak stress increases by 170.3% and the elastic modulus increases by 201.1%. The energy evolution mechanism transitions from progressive damage with gradual energy dissipation at low roughness to a three-stage mode at high roughness, characterized by initial frictional energy dissipation, intermediate energy storage, and rapid elastic energy release and dissipated energy increase near failure. DIC results further reveal that increasing interface roughness suppresses interfacial shear slip and promotes tensile-dominated strain localization, whereas excessive roughness may induce local stress concentration around asperities and increase the tendency toward abrupt post-peak instability, the failure mode changes from mixed tensile–shear failure with obvious interfacial slip to tensile-dominated failure.

## 1. Introduction

In the field of underground engineering, sandstone and concrete often jointly bear loads in the form of composite structures, and typical applications include mountain tunnels, hydraulic tunnels, underground gas storage caverns and underground coal mine projects [1,2,3,4,5,6]. In such geological-engineering composite structures, the bedrock and concrete lining are directly combined to form a collaborative stress-bearing system. However, due to the essential differences in the mechanical properties of the two types of materials, significant stress concentration and deformation incompatibility are likely to occur in the interface area, which may induce the initiation and propagation of microcracks, and even lead to structural leakage and instability failure [7,8,9]. Therefore, it is of great theoretical significance and engineering value to deeply reveal the mechanical behavior and failure mechanism of sandstone–concrete composites and clarify the regulation mechanism of interface characteristics on the overall bearing capacity for optimizing the design of composite structures and ensuring the safety and stability of underground engineering.

In recent years, scholars at home and abroad have carried out a large number of research works on the mechanical properties of rock-concrete composites. Existing studies mainly focus on the peak strength, elastic modulus and failure characteristics of the composites under loading conditions such as uniaxial compression, triaxial compression and direct shear, and explore the influence laws of factors such as rock type, concrete strength, interface inclination angle and curing conditions [10,11,12]. For example, Yuan et al. [13] systematically investigated the mechanical properties and energy evolution characteristics of sandstone–concrete composites. Dong et al. [14] studied the biaxial compression tests of the filling body-rock composite under different stress conditions. In terms of interface property research, interface roughness is generally considered as the key factor controlling the mechanical behavior of the rock-concrete composite [15]. Zhao et al. [16] quantified the synergistic effect of roughness and confining pressure through systematic triaxial compression tests, revealing the objective existence of the critical *JRC* threshold and the geometric anchoring mechanism of roughness regulating the crack propagation path through strain localization. Zheng et al. [17] independently developed a rock-concrete interface shear-seepage test system and clarified the five-stage evolution characteristics of the interface during the shear-seepage process under the hydro-mechanical coupling condition. Wang et al. [18] studied the mechanical behavior and failure process of the rock-concrete bimaterial disk under dynamic tensile loading. Badika et al. [19] proposed a new method to estimate the peak shear strength of the concrete-rock interface. Zhao et al. [20] found that increasing interface roughness can significantly enhance the bearing capacity of the rock-concrete structure. Chen et al. [21] systematically investigated the mechanical response and interfacial damage evolution of rock–concrete composite structures. In terms of testing and characterization methods, acoustic emission (AE) and digital image correlation (DIC) techniques have been widely used to monitor damage evolution and fracture processes in rock, concrete, and composite materials [22,23,24]. Meanwhile, recent studies on rock–concrete interfaces have shown that interface roughness significantly affects fracture behavior, crack propagation, and interfacial damage development under complex loading conditions [25]. These findings indicate that combining mechanical testing with sensor-based monitoring and deformation-field characterization is an effective approach for revealing the damage evolution mechanism of rock–concrete composite structures. However, most existing studies have focused on binary systems composed of natural rock and ordinary concrete or backfill materials, while the influence of concrete matrix composition, such as the incorporation of mineral admixtures, on the interfacial performance of rock–concrete composites remains insufficiently understood. In fact, changes in the concrete matrix may significantly affect the mechanical behavior of the interfacial transition zone, but this factor has not been fully considered in previous studies.

It is worth noting that in the context of solid waste resource utilization and low-carbon development, using industrial solid wastes such as fly ash, slag, steel slag, and marble waste powder as mineral admixtures to partially replace cement to prepare green concrete has become an important technical path to reduce the carbon footprint of projects. Akbulut et al. [26] systematically reviewed the application of Class C and Class F fly ash as supplementary cementitious materials in concrete; Moula et al. [27] studied the feasibility of using granulated blast furnace slag to replace cement to prepare ultra-high performance concrete; Yüksel et al. [28] reviewed the application progress of steel slag in the construction industry. Among them, marble waste powder, as the main by-product of stone processing, has an annual emission of up to several million tons in Guangxi alone. Its open-air stacking or landfill will cause environmental problems such as dust pollution, soil compaction, and water body alkalization. Using it as a supplementary cementitious material to replace cement can significantly reduce the amount of clinker used and carbon emissions while realizing the resource utilization of solid waste, with both environmental and economic benefits. Prakash et al. [29] reviewed the sustainability of marble powder as a continuous cement replacement material; Wang et al. [30] studied the influence law of marble waste powder with different fineness and dosages on the mechanical properties of cement-based materials. Singh et al. [31] and Aliabdo et al. [32] further verified its application feasibility from the perspectives of long-term durability and comprehensive performance. The above research fully verified the technical feasibility of using marble waste powder as an auxiliary cementitious material to partially replace cement, providing an important basis for promoting the development of rock-concrete composites towards the direction of green and low carbon. However, current research on marble waste powder concrete mainly focuses on the working performance, mechanical strength, etc., at the single material level. For the composite formed by marble waste powder concrete and rock, its mechanical and interfacial characteristics lack systematic research.

In this study, sandstone–marble waste powder concrete composites with different interface roughness coefficients were prepared and tested under uniaxial compression. Acoustic emission and digital image correlation techniques were synchronously applied to monitor internal damage activity. The objective was to reveal the effects of interface roughness on compressive strength, energy evolution, AE response, and failure modes, thereby providing experimental evidence for sensor-based damage monitoring and in-terface design of green rock–concrete composite structures.

## 2. Experimental Materials and Procedures

### 2.1. Experimental Materials

**Figure 1:**
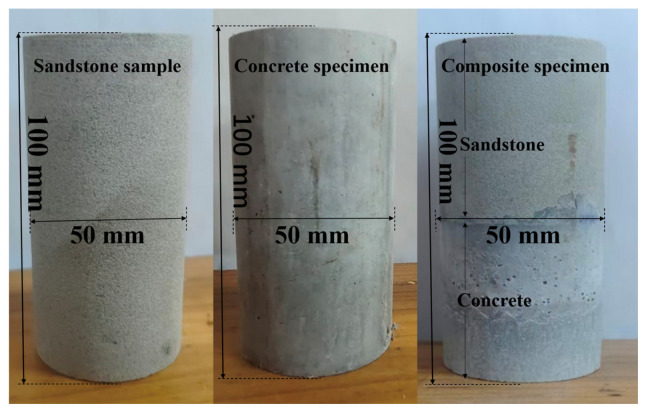
Three types of specimens used in this study.

A total of three types of specimens were prepared in this study, namely sandstone, concrete and sandstone–marble waste powder concrete composites with different roughness. All specimens were processed into cylindrical structures with a diameter of 50 mm and a height of 100 mm according to the standards of the International Society for Rock Mechanics (ISRM) (as shown in Figure 1), and the specific preparation process is described as follows.

#### 2.1.1. Preparation of Sandstone Specimens

Core samples were drilled from the same parent rock in Zigong, Sichuan. After end polishing, a small number of standard cylindrical specimens were processed. The mineral composition of the sandstone was determined by X-ray diffraction (XRD) analysis, which revealed that the sandstone mainly consists of albite (26.2 wt%), quartz (46.5 wt%), microcline (9.5 wt%), calcite (5.8 wt%), chamosite (3.1 wt%), dolomite (2.3 wt%), and muscovite (6.6 wt%). The bulk density and porosity of the sandstone were 2.31 g/cm^3^ and 13%, respectively. The UCS and equivalent elastic modulus were approximately 41 MPa and 7.72 GPa, respectively. All sandstone samples used in the tests originated from this parent rock, aiming to reduce the inherent heterogeneity of sandstone in terms of structure, mineral composition and pore characteristics, thereby reducing its interference with the test results.

#### 2.1.2. Preparation of Concrete Specimens

**Figure 2:**
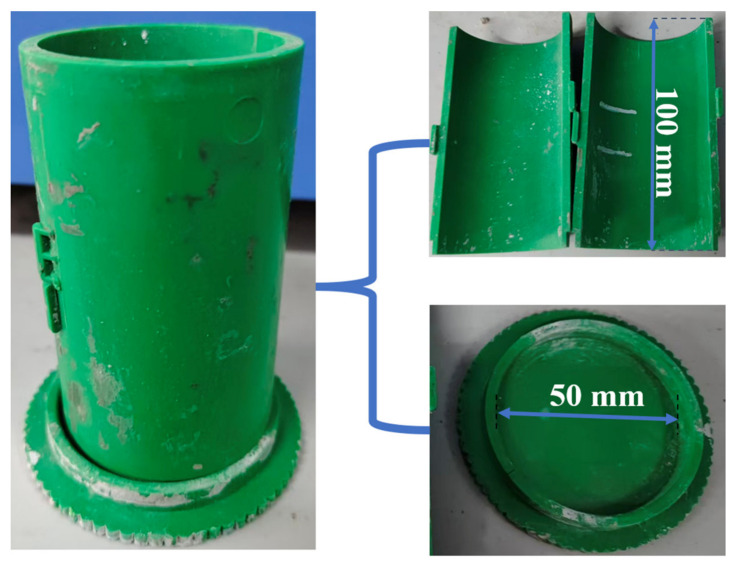
Mold used for specimen preparation.

The cement used in this study was P.O. 42.5 ordinary Portland cement. The mineral powder was S95 ground granulated blast furnace slag (GGBFS). The marble waste powder was obtained from Hezhou, Guangxi, China. The marble waste powder was ground for 30 min, and the Blaine specific surface area was 732 m^2^/kg. XRF results showed that the marble waste powder mainly contained LOI 43.6%, CaO 47.62%, SiO_2_ 1.92%, Al_2_O_3_ 0.36%, Fe_2_O_3_ 0.57%, and MgO 6.29%. XRD results indicated that calcite and dolomite were the dominant crystalline phases. Therefore, in this study, marble waste powder mainly acted as a mineral filler with nucleation effects. The concrete matrix was prepared using cement, GGBFS, marble waste powder, water, polycarboxylate superplasticizer, and PE fibers. In the binder system, cement, GGBFS, and marble waste powder accounted for 60 wt%, 15 wt%, and 25 wt%, respectively. Correspondingly, in the total mixture, cement, GGBFS, marble waste powder, superplasticizer, and water accounted for 49.07 wt%, 12.20 wt%, 20.45 wt%, 1.64 wt%, and 16.36 wt%, respectively. The water-to-binder ratio was 0.20. PE fibers were added at 1 vol%, with a length of 12 mm, a diameter of 25 μm, an aspect ratio of approximately 480, an elastic modulus of 122 GPa, a tensile strength of 3100 MPa, and a density of approximately 0.98 g/cm^3^. The preparation process of concrete specimens is as follows: After dry mixing the cement, GGBFS, and marble waste powder, water and superplasticizer were added and mixed until a homogeneous matrix was obtained. PE fibers were then gradually dispersed into the mixture to avoid agglomeration. The fresh mixture was cast into cylindrical molds (Figure 2), vibrated for compaction, sealed, cured for 24 h, demolded, and then cured under standard conditions until 28 d. Before testing, the specimens were cut, ground, dried at 60 °C for 12 h, and cooled to room temperature. The specimens exhibited a peak stress exceeding 80 MPa at 28 days and an equivalent elastic modulus of 8.147 GPa.

#### 2.1.3. Preparation of Sandstone–Marble Waste Powder Concrete Composite Specimens

**Figure 3:**
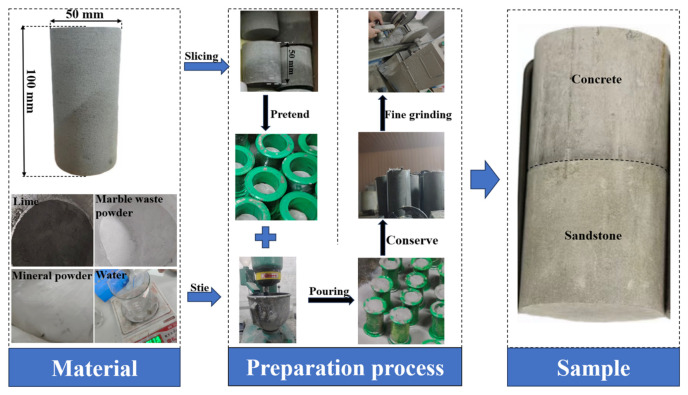
Preparation process of sandstone–marble waste powder concrete composite specimens.

The composite specimens were prepared by placing the processed sandstone specimen at the bottom of the cylindrical mold and casting the fresh concrete matrix onto the sandstone surface. The molds were vibrated for compaction, sealed, cured for 24 h, demolded, and then cured under standard conditions until 28 d. Before testing, the end faces of the composites were cut and ground to ensure dimensional consistency, especially near the interface region (Figure 3).

#### 2.1.4. Preparation of Sandstone–Marble Waste Powder Concrete Composite Specimens with Roughness

**Figure 4:**
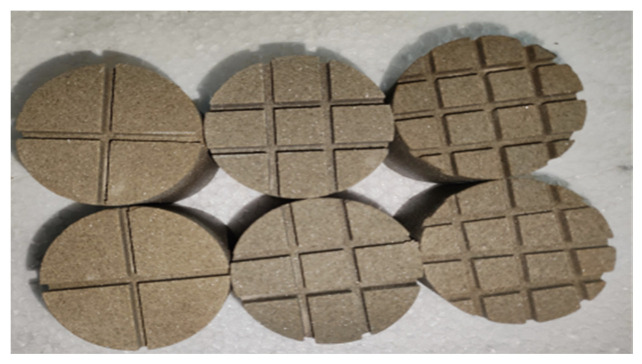
Sandstone interfaces with different roughness levels.

**Figure 5:**
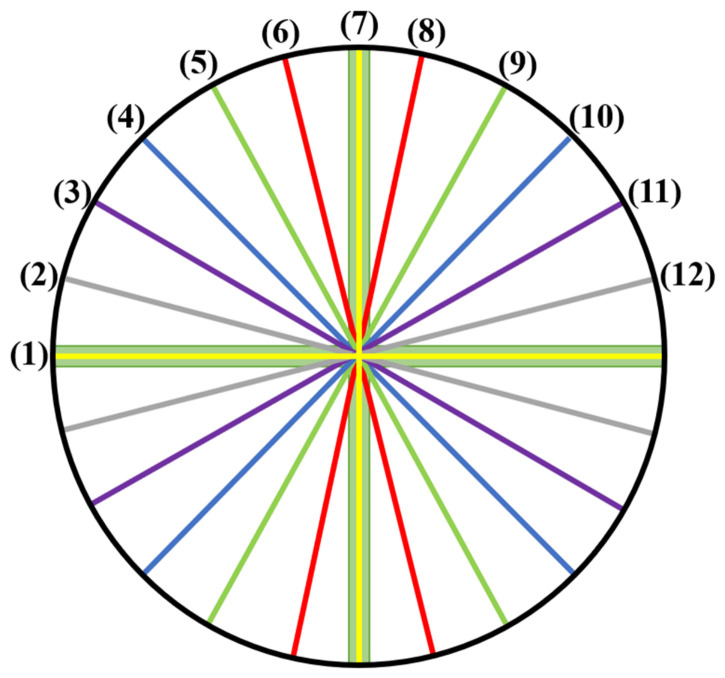
Schematic diagram of the rough surface and measurement lines.

In this study, the interface roughness was quantitatively characterized by the joint roughness coefficient (*JRC*). Four sandstone interfaces with different roughness morphologies were prefabricated. The characteristic depth and width of the roughness structures were 2 mm and 4 mm, respectively, as shown in Figure 4. For the four roughness levels, the subsequent pouring and curing processes of the sandstone–concrete composite specimens were consistent with those described above. To quantify the interface roughness, 12 measurement lines passing through the center of the circular interface were uniformly arranged on the cross-section of the specimen, as shown in Figure 5. The three-dimensional morphology of the rough surface was characterized using the geometric features of these 12 contour lines. Each contour line was treated as a two-dimensional roughness profile, and the corresponding two-dimensional roughness coefficient, was calculated using the empirical Equation (1) [33]:

(1) $$JRC^{2D}=32.69+32.98\log_{10}Z_{2}$$

The parameter $Z_{2}$ is given by Equation (2).

(2) $$Z_{2}={\left[\frac{1}{m{\left(\Delta x\right)}^{2}}{\sum_{j=1}^{m}\left(f_{j+1}-f_{j}\right)}^{2}\right]}^{1/2}$$

where Δx is the interval distance between the sampling points on each contour line, Δx=0.25 mm; $f_{j+1}$ and $f_{j}$ are the vertical coordinates of two adjacent sampling points, and the vertical difference between them is $f_{j+1}-f_{j}$; m is the number of sampling points on each contour line, which depends on the diameter D of the circular rough surface, that is, m=1+int(D/Δx); the roughness coefficient of the interface is determined as the average of the *JRC* values of the 12 contour lines, that is:

(3) $$JRC=\frac{1}{12}\sum_{j=1}^{12}(JRC^{2D})$$

where JRC is the joint roughness coefficient; $JRC^{2D}$ is the roughness coefficient of each contour line.

The calculated *JRC* values for the four interface roughness levels were 0, 7.84, 17.99, and 20.79. For convenience, the corresponding sandstone–concrete composite specimens were denoted as *J*_1_, *J*_2_, *J*_3_, and *J*_4_, respectively.

### 2.2. Test Methods

**Figure 6:**
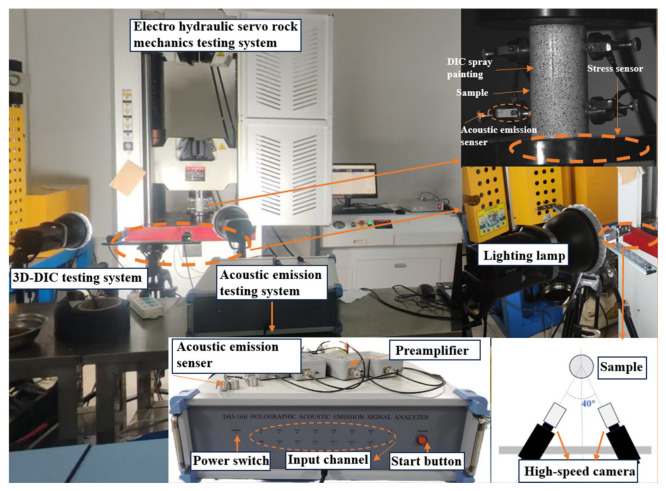
Combined test setup: electro-hydraulic servo rock mechanics testing machine, AE system, and 3D-DIC system.

For each group, three replicate specimens were prepared and tested under uniaxial compression. The reported mechanical parameters were calculated from the valid specimens. The test system consisted of an electro-hydraulic servo rock mechanics testing machine, an AE monitoring system, and a 3D-DIC system (Figure 6). The relevant experimental procedures are as follows: (I) Uniaxial compression loading scheme: An MTS815.04 electro-hydraulic servo testing machine (MTS Systems Corporation, Eden Prairie, MN, USA) was used under displacement control at a rate of 0.3 mm/min, with a maximum displacement of 5.0 mm, and the test was automatically terminated after specimen failure. (II) Acoustic emission monitoring scheme: A PCI-II multi-channel acoustic emission system (Physical Acoustics Corporation (PAC) (now part of MISTRAS Group), Princeton Junction, NJ, USA) was used to record parameters such as counts, amplitude, and duration in real time. A total of four AE sensors were used during the tests. To minimize occlusion of the DIC observation area, the sensors were positioned slightly off the front face. The acquisition threshold was 35 dB, and the sampling rate was 3 MHz. In addition to AE hits and cumulative AE energy, stage-based AE statistics and RA–AF parameters were calculated as supplementary sensing indicators. However, due to the limitation of the present AE monitoring configuration and exported dataset, b-value/Ib-value evolution, detailed frequency-distribution analysis, and reliable AE source localization were not conducted in this study. (III) Digital image correlation test scheme: A VIC-3D system (Correlated Solutions, Inc., Columbia, SC, USA) was used with two high-speed cameras symmetrically set at an angle of approximately 40°, triggered synchronously, at a frame rate of 40 fps. The specimen surface was first sprayed with three coats of white primer, and then black paint was applied to create a speckle pattern with an average speckle size of about 0.55 mm for the measurement of full-field strain and displacement fields.

## 3. Results

### 3.1. Stress–Strain Full Curve Analysis

The elastic modulus is defined as the ratio of stress to the corresponding strain, as shown in Equation (4). In this study, the elastic modulus was calculated from the approximately linear segment around 50% of the peak stress. This approach is commonly used in rock mechanics to obtain a representative tangent or secant modulus from the relatively linear portion of the stress–strain curve. It can reduce the influence of initial pore compaction and end-contact adjustment at low stress levels and avoid the strong nonlinear damage accumulation near peak stress.

(4) $$E=\frac{\sigma }{\varepsilon }$$

where E represents the elastic modulus; σ is the stress; and ε is the strain.

**Figure 7:**
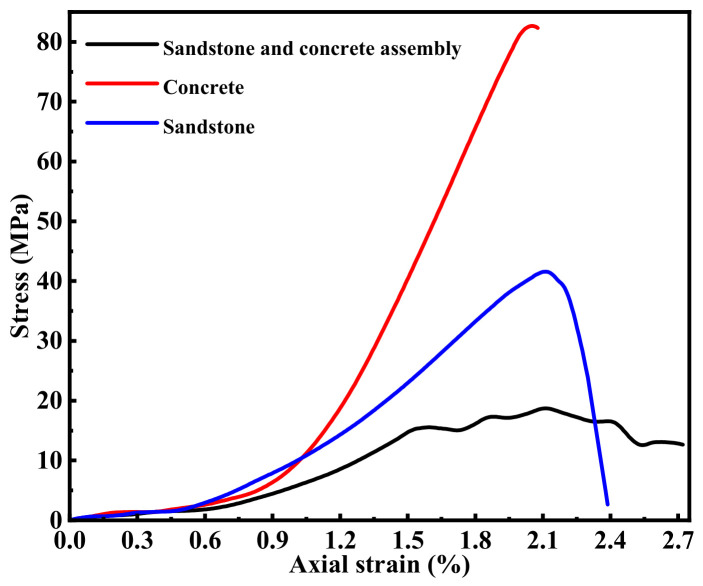
Stress–strain curves of concrete, sandstone, and sandstone–concrete composite specimens.

Figure 7 shows the stress–strain curves of the concrete, sandstone, and sandstone–concrete composite specimens. The concrete specimen exhibits the highest peak stress among the three specimen types. After a short initial compaction stage, the stress increases rapidly with axial strain and reaches a peak value exceeding 80 MPa. The sandstone specimen shows a lower peak stress of approximately 41 MPa, followed by a rapid stress drop after the peak, indicating a significant loss of bearing capacity. In contrast, the sandstone–concrete composite specimen exhibits the lowest peak stress, approximately 18 MPa, and its stress–strain curve shows noticeable fluctuations during loading. After the peak stress, the stress decreases gradually and maintains a certain residual bearing capacity over a larger strain range. According to Equation (4), the equivalent elastic moduli of the marble waste powder concrete and sandstone specimens were 8.147 GPa and 7.7232 GPa, respectively. These values indicate that the two materials have comparable but not identical stiffness characteristics, and this stiffness mismatch may contribute to stress redistribution and local damage development at the sandstone–concrete interface. Overall, compared with the single sandstone and concrete specimens, the sandstone–concrete composite shows a lower peak stress and more pronounced post-peak fluctuation, indicating that the interface controls stress transfer and local damage evolution. The stiffness mismatch between sandstone and concrete promotes stress redistribution near the interface, thereby weakening the overall load-bearing capacity of the composite.

**Figure 8:**
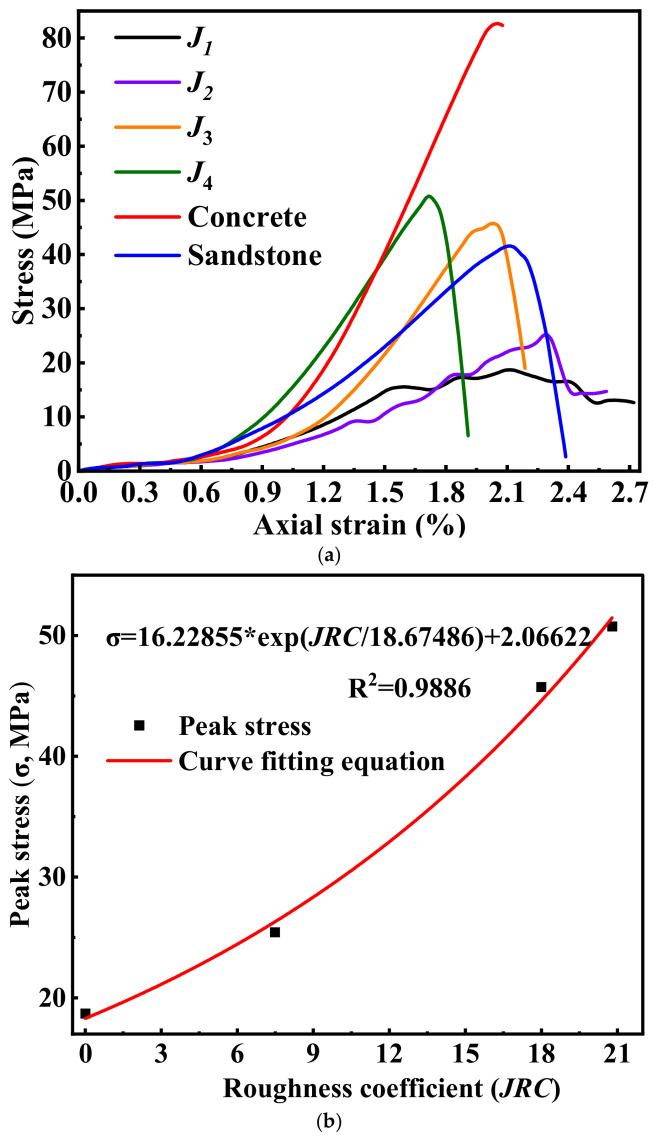

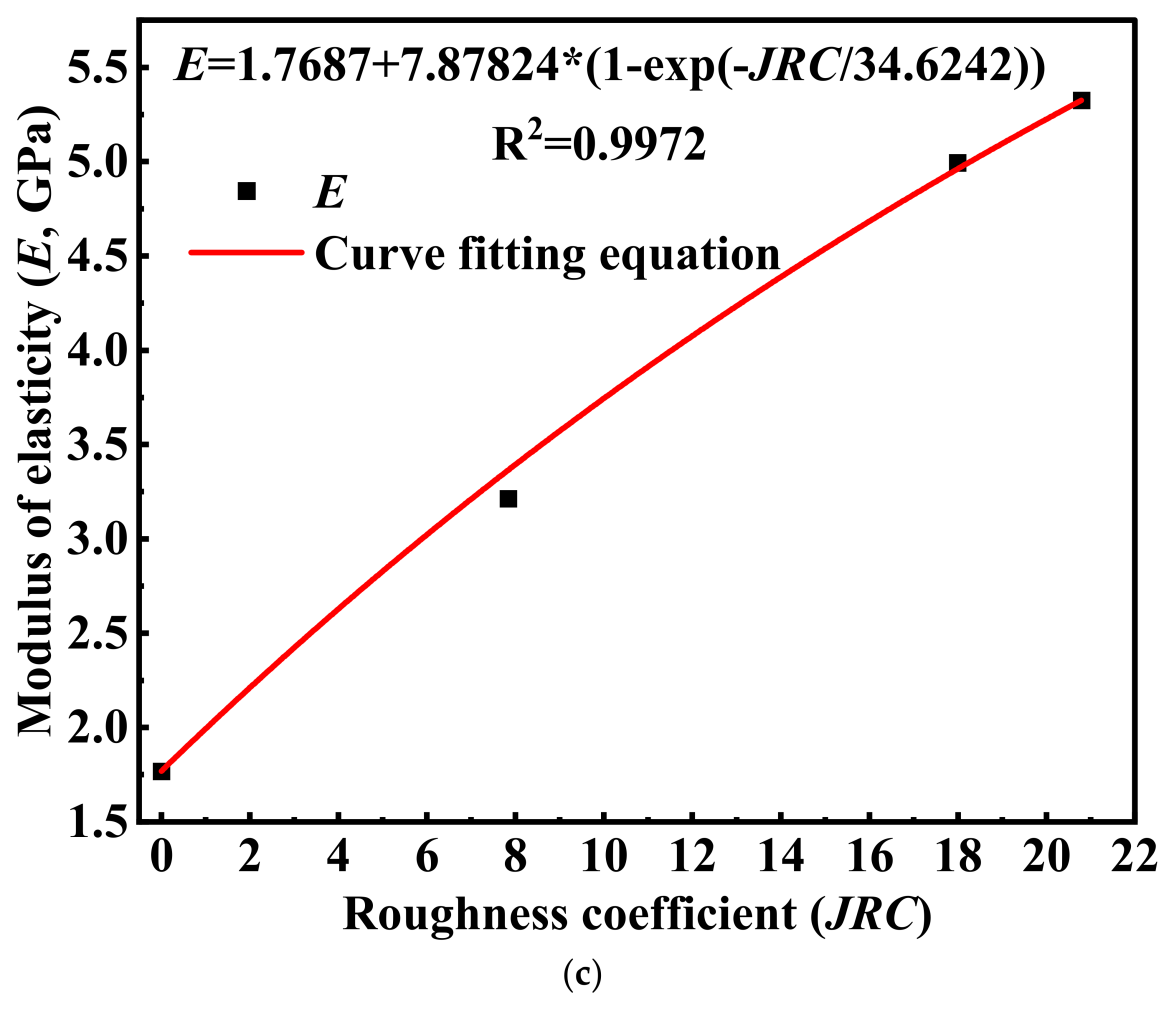
Uniaxial compression test results of specimens with different roughness: (**a**) stress–strain curves; (**b**) peak stress; (**c**) elastic modulus.

Uniaxial compression tests were performed on six groups of specimens, including sandstone, concrete, and four sandstone–marble waste powder concrete composite groups with different *JRC* values, and their stress–strain curves are presented in Figure 8a. The peak stress increases progressively from *J*_1_ to *J*_4_, indicating a clear roughness-dependent strengthening effect. All specimens exhibit an initial compaction stage, followed by an approximately linear stage and a post-peak stress drop. The mechanical response varies systematically with interface roughness.

As shown in Figure 8b, the peak stress increases from 18.5 MPa to 50.0 MPa as *JRC* increases from 0 to 20.79, corresponding to an overall increase of 170.3%. The growth rate gradually decreases at higher roughness, suggesting that the strengthening effect tends to approach saturation. These results indicate that increasing interface roughness can significantly enhance the compressive strength of the composite, mainly due to strengthened mechanical interlocking and frictional resistance at the sandstone–concrete interface. The 170.3% increase should be interpreted as an increase in the apparent bearing capacity of the composite rather than an increase in the intrinsic strength of sandstone or concrete. With increasing *JRC*, the real contact area, asperity interlocking, frictional resistance, and interface constraint are enhanced, which suppresses interfacial slip and delays premature debonding. For the *J*_4_ specimen, the high-roughness interface promotes more effective load sharing between sandstone and marble waste powder concrete. Therefore, its apparent peak stress can exceed that of the sandstone specimen, although it remains lower than that of the concrete specimen.

As shown by the fitting curve of the elastic modulus in Figure 8c, the elastic modulus also exhibits a stable upward trend with increasing interface roughness, without obvious fluctuations or decreases. The fitted curve shows that the equivalent elastic modulus increases with *JRC*, indicating that higher roughness improves interfacial bonding stiffness and stress-transfer efficiency. This enhancement is mainly attributed to improved interfacial bonding, stronger mechanical interlocking, more efficient load transfer, and reduced relative slip at the interface, thereby improving the overall mechanical performance of the composite.

### 3.2. Analysis of Energy Evolution and Dissipation Mechanisms

Energy analysis was used to characterize the storage and dissipation processes of the composites during uniaxial compression. Assuming no heat exchange with the surroundings during loading, the external work applied by the testing machine is mainly converted into elastic energy and dissipated energy. The relationship among the total energy, elastic energy, and dissipated energy of the specimen is:

(5) $$U=U_{d}+U_{e}$$

The formula for calculating the total energy is:

(6) $$U_{d}=\int_{0}^{\varepsilon }\sigma d\varepsilon$$

where ε is the axial strain; σ is the axial stress; the formula for calculating the elastic energy is simplified to:

(7) $$U_{e}=\frac{\sigma^{2}}{2E}$$

where E is the elastic modulus.

From Equations (5) and (7), the formula for calculating the dissipated energy can be obtained as:

(8) $$U_{d}=U-U_{e}=\int_{0}^{\varepsilon }\sigma d\varepsilon -\frac{\sigma^{2}}{2E}$$

This macroscopic energy partition method has been widely adopted in rock mechanics to evaluate energy storage, energy dissipation, and damage evolution under compression loading. Previous studies have applied similar stress–strain-curve-based energy analysis to investigate the energy storage and dissipation laws of rocks under uniaxial compression, cyclic loading–unloading compression, and coupled static–dynamic compression [34,35,36,37,38,39]. In these studies, the total input energy density is generally obtained by integrating the stress–strain curve, the recoverable elastic strain energy is estimated using a strain-energy formula derived from elastic theory, and the dissipated energy is calculated from energy conservation. 

**Figure 9:**
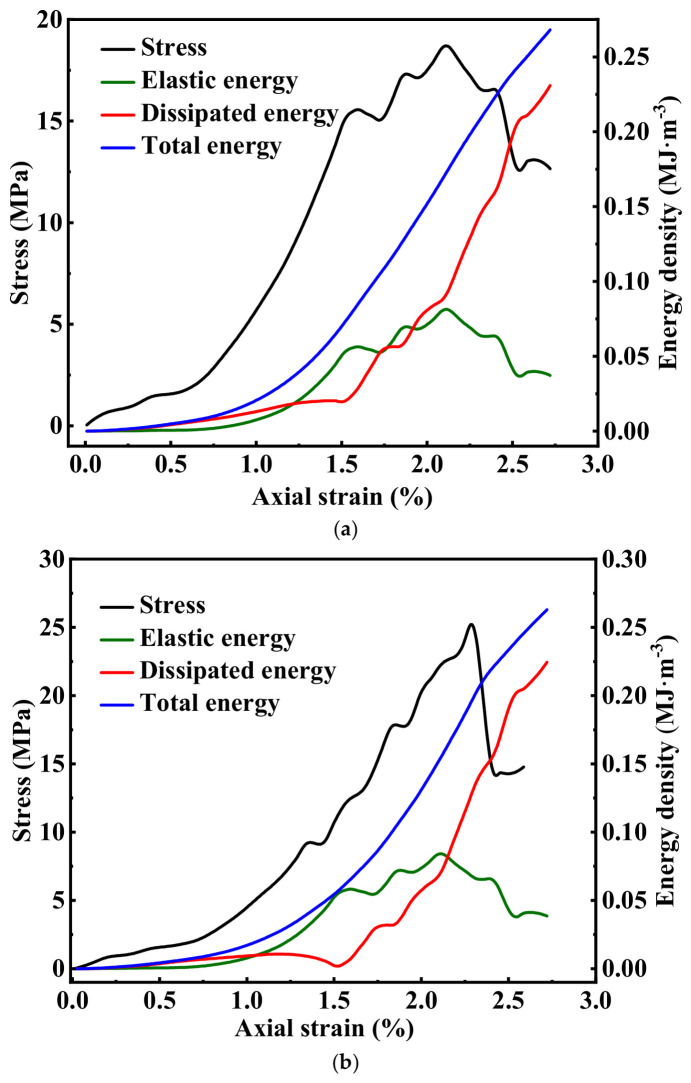

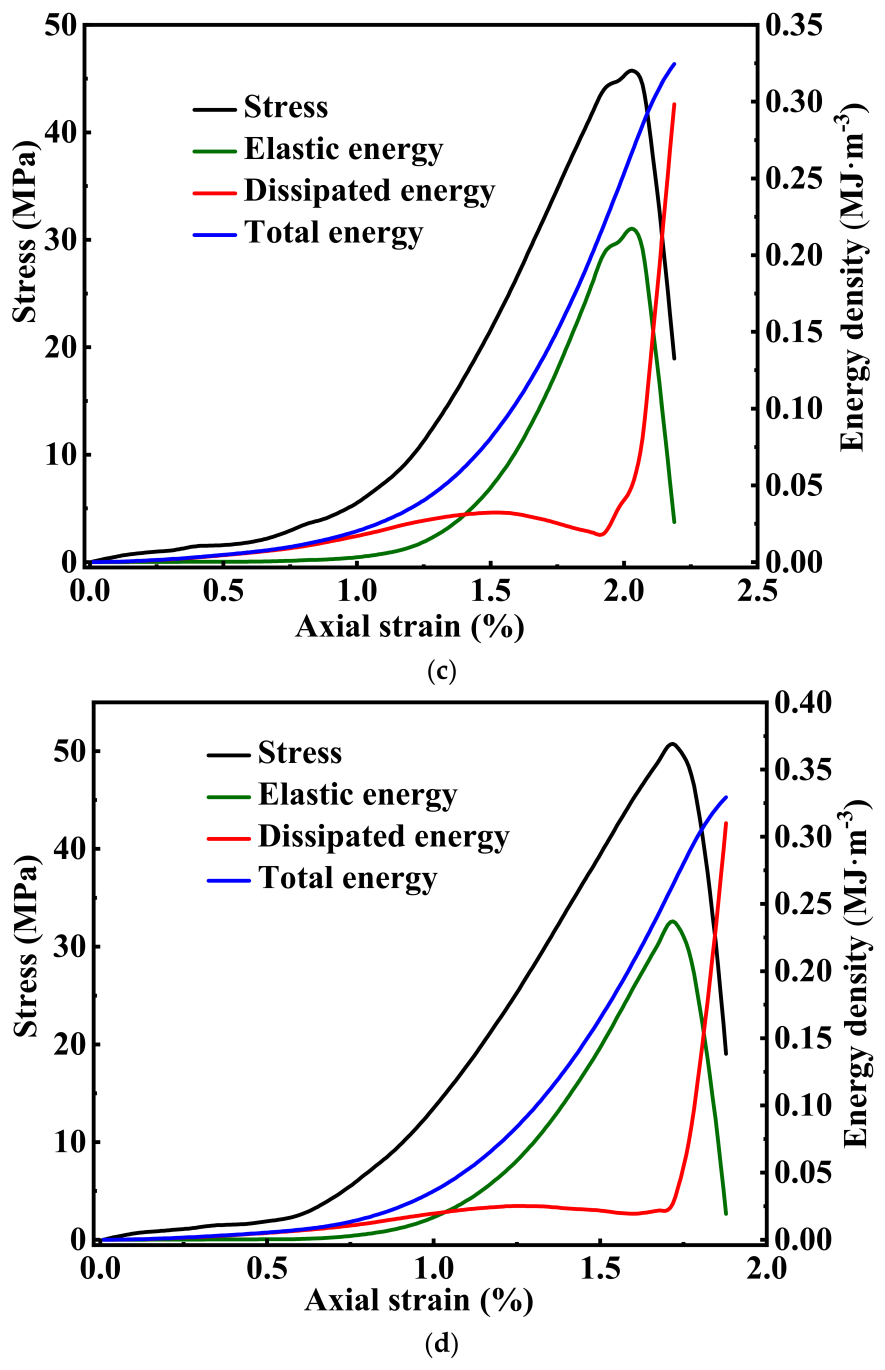
Strain–energy density curves of specimens with different interface roughness under uniaxial compression: (**a**) *J*_1_; (**b**) *J*_2_; (**c**) *J*_3_; (**d**) *J*_4_.

Figure 9 shows that interface roughness strongly affects the balance between elastic energy storage and dissipated energy growth. In low-roughness specimens, especially *J*_1_ and *J*_2_, the gradual increase in dissipated energy indicates progressive damage accumulation and relatively ductile failure. In contrast, *J*_3_ and *J*_4_ store more elastic energy before peak stress, followed by a sharp decrease in elastic energy and rapid increase in dissipated energy near failure. This transition suggests that higher roughness enhances pre-peak energy storage through stronger interfacial interlocking, but also promotes abrupt energy release once interfacial debonding and crack coalescence occur.

The energy evolution patterns of *J*_1_–*J*_4_ differ systematically (Figure 9). The elastic energy density increases with *JRC*, while the dissipated energy density shows a more abrupt rise near failure in high-*JRC* specimens.

### 3.3. Acoustic Emission Characteristics Under Different Interface Roughness Levels

**Figure 10:**
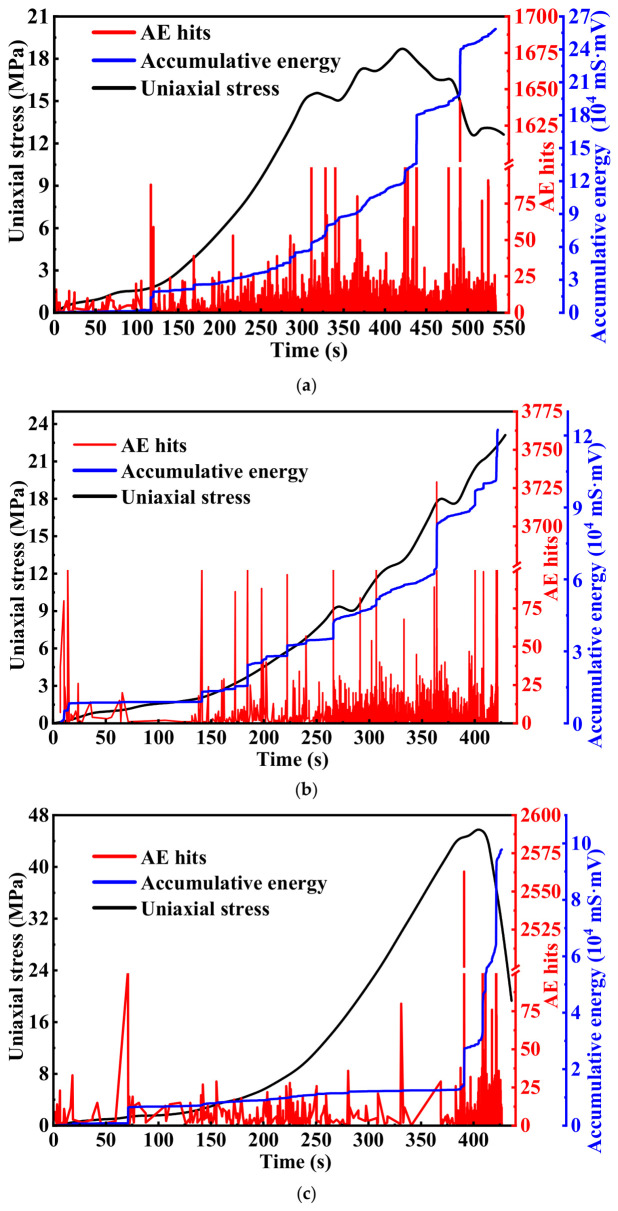

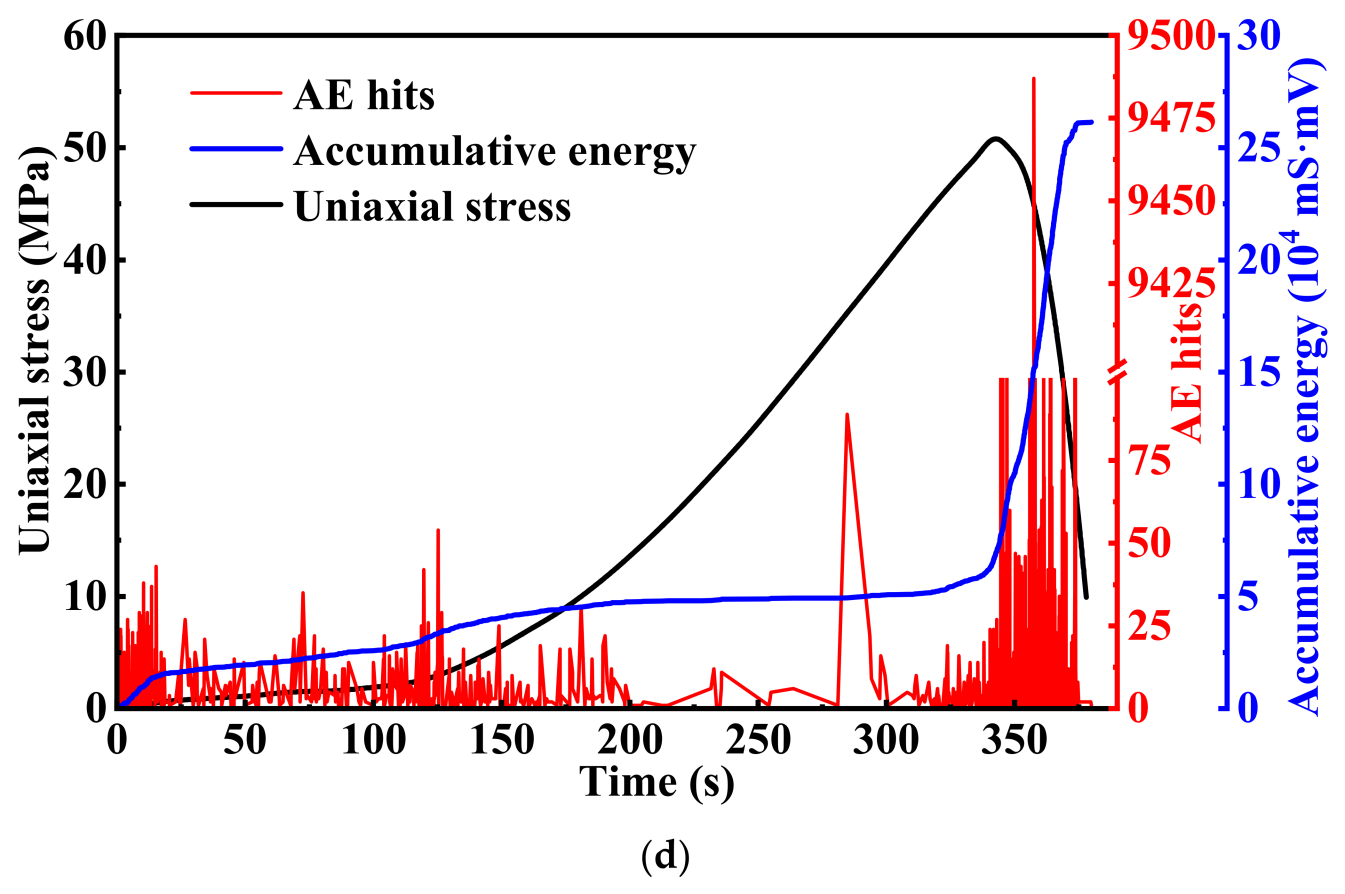
Stress–acoustic emission response curves of specimens with different interface roughness under uniaxial loading: (**a**) *J*_1_; (**b**) *J*_2_; (**c**) *J*_3_; (**d**) *J*_4_.

Figure 10 shows the stress–AE response curves of sandstone–concrete composite specimens with different interface roughness under uniaxial compression. For the *J*_1_ specimen, AE signals appear throughout almost the whole loading process, and the accumulative energy increases gradually with several step-like rises. This indicates that microcrack initiation and propagation occur progressively during loading. Near the peak stress and post-peak stage, AE hit activity becomes more intense, while the stress curve shows obvious fluctuation and degradation, suggesting continuous damage accumulation and progressive failure. For the *J*_2_ specimen, the AE activity is relatively weak in the early stage, followed by intermittent AE hits as the stress increases. The accumulative energy increases in a stepwise manner before peak stress and rises more rapidly near failure, indicating that crack development becomes more active in the later loading stage. Compared with *J*_1_, the AE activity of *J*_2_ is less continuously distributed and is more closely related to the later-stage damage evolution. For the *J*_3_ specimen, AE activity remains at a relatively low level over a long period during the early and middle loading stages, and the accumulative energy increases only slightly. When the stress approaches the peak value, AE hits increase sharply, accompanied by a sudden rise in cumulative energy. This response indicates that a large amount of damage is released within a short time, corresponding to rapid crack coalescence and unstable failure. For the *J*_4_ specimen, the AE response shows a more pronounced abrupt characteristic. Although scattered AE events occur in the early and middle stages, the accumulative energy remains relatively stable before peak stress. Near failure, both AE hits and cumulative energy increase sharply, and the stress drops rapidly after reaching the peak. This indicates that the high-roughness interface enhances mechanical interlocking and elastic energy accumulation before failure, but once interfacial debonding and crack propagation occur, the stored energy is rapidly released, resulting in sudden instability. The stage-based AE response characteristics for all specimens are summarized in Table 1. As shown in Table 1, with increasing interface roughness, the low-activity stage prolongs (from 0–90 s for *J*1 to 180–330 s for *J*4), while the burst stage becomes shorter and more intense (from 430–520 s for *J*1 to 345–365 s for *J*4). Correspondingly, the damage feature transitions from progressive damage (*J*1) to sudden instability (*J*4). This stage-based transition quantitatively confirms the evolution from continuous damage accumulation to localized unstable failure as interface roughness increases.

As shown in Table 2, *J*_3_ and *J*_4_ exhibit higher mean RA values and lower mean AF values than *J*_1_ and *J*_2_, indicating that the increase in interface roughness enhances the contribution of frictional sliding and mixed tensile–shear microcracking. Therefore, the RA–AF results provide supplementary AE evidence for the transition from progressive damage to localized unstable failure. To further quantify the AE characteristics, RA–AF analysis was performed based on the AE hit parameters. The RA value was calculated as the ratio of rise time to amplitude, while the AF value was calculated as the ratio of AE counts to duration. In general, low RA and high AF values are associated with tensile cracking, whereas high RA and low AF values indicate a stronger shear or frictional sliding component. The calculated mean RA values of *J*_1_, *J*_2_, *J*_3_, and *J*_4_ were 0.1032, 0.1031, 0.1731, and 0.1627 μs/mV, respectively, and the corresponding mean AF values were 236.09, 238.41, 157.77, and 162.03 kHz, respectively. Compared with *J*_1_ and *J*_2_, *J*_3_ and *J*_4_ exhibited higher RA values and lower AF values, indicating that increasing interface roughness enhanced local frictional sliding and mixed tensile–shear microcracking due to asperity interlocking and stress concentration. This result is consistent with the energy evolution and DIC observations, showing that high interface roughness improves the load-bearing capacity but also promotes localized damage accumulation and abrupt post-peak instability.

Overall, with increasing interface roughness, the peak stress of the sandstone–concrete composite increases significantly, indicating that a rougher interface improves the interfacial contact condition, mechanical interlocking effect, and load-bearing capacity. However, the AE characteristics do not show a simple monotonic increase with roughness. Instead, the damage evolution changes from relatively continuous and progressive AE activity in *J*_1_ and *J*_2_ to more concentrated and abrupt AE release in *J*_3_ and *J*_4_. In particular, the *J*_4_ specimen exhibits the strongest late-stage AE burst, reflecting the most concentrated damage release. This result is consistent with the energy evolution analysis: higher interface roughness improves the energy storage capacity of the composite, but also increases the suddenness of post-peak failure. Therefore, interface roughness not only enhances the compressive strength of sandstone–concrete composites, but also significantly affects the damage evolution process and failure instability characteristics.

### 3.4. Analysis of Crack Propagation and Failure Modes Based on DIC

**Figure 11:**
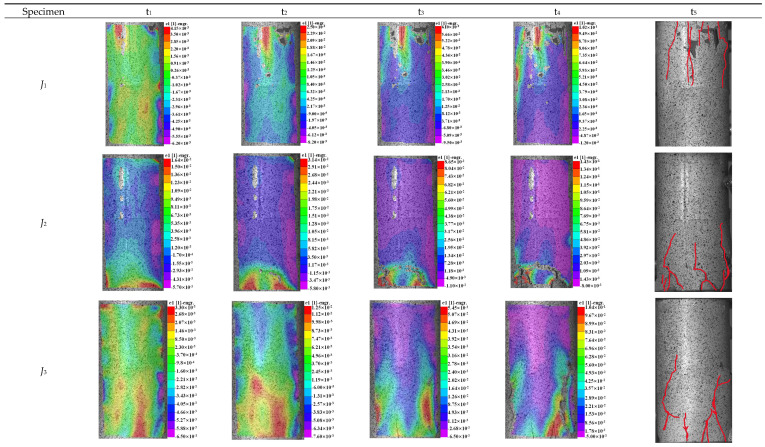

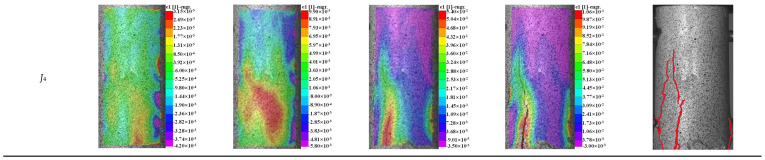
Horizontal strain evolution of specimens with different roughness coefficients obtained by DIC.

Figure 11 presents the evolution of the horizontal strain field of sandstone–concrete composite specimens with different interface roughness during uniaxial compression. For the *J*_1_ specimen, the strain field is relatively scattered at the initial loading stage, and localized tensile strain gradually develops in the upper part of the specimen as loading proceeds. At the later stage, the tensile strain concentration becomes more pronounced, accompanied by obvious strain redistribution in the surrounding regions, indicating that crack propagation is coupled with local shear slip. Therefore, the failure of *J*_1_ can be characterized as tensile-dominated mixed tensile–shear failure. For the *J*_2_ specimen, tensile strain mainly concentrates near the lower part and the interface-related local zones, while the overall strain field remains relatively continuous during loading. The peak tensile strain is higher than that of the other specimens, suggesting better deformation coordination and stronger tensile deformation capacity at this roughness level. Compared with *J*_1_, the shear-related strain localization is weakened, and the failure process is mainly controlled by tensile strain development. For the *J*_3_ and *J*_4_ specimens, the strain fields exhibit more obvious spatial heterogeneity from the early loading stage, with alternating tensile and compressive strain zones, indicating that the increase in interface roughness enhances local stress adjustment and interfacial interlocking. With increasing load, tensile strain localization gradually develops from the lower and side regions and becomes more concentrated before failure. In particular, the *J*_4_ specimen shows more distinct localized strain bands, reflecting stronger mechanical interlocking and more significant local stress concentration at the high-roughness interface.

To quantitatively characterize the strain localization behavior, the maximum principal strain e1max (In this study, e1 denotes the first principal strain obtained from the DIC analysis) and the surface-averaged principal strain were extracted from the exported DIC full-field data at the peak-stress moment, and the strain concentration factor (SCF) was calculated as the ratio of e1max to the surface-averaged e1.

As summarized in Table 3, the SCF decreases monotonically from 3.85 (*J*_1_) to 2.07 (*J*_4_) with increasing interface roughness. This indicates that a rougher interface promotes more uniform strain distribution and suppresses extreme localized deformation. Among all specimens, *J*_2_ exhibits the highest e1max (0.143) and a substantially reduced SCF (2.12) compared with *J*_1_ (3.85), suggesting that moderate roughness provides the best deformation coordination, allowing the specimen to accommodate larger local tensile strain without severe strain localization. For *J*_3_ and *J*_4_, the SCF further decreases to 2.11 and 2.07, respectively, implying that the strain-uniformization effect tends to saturate at high roughness levels. These quantitative DIC results are consistent with the observed transition in failure modes from tensile-dominated mixed tensile-shear failure (*J*_1_) to tensile-dominated failure (*J*_2_–*J*_4_), as discussed in the qualitative strain-field evolution above.

Overall, increasing interface roughness suppresses the shear-slip tendency observed in *J*_1_ and promotes a transition toward tensile-dominated failure. However, this effect is not monotonically beneficial. Moderate roughness, represented by *J*_2_, improves deformation coordination and tensile strain capacity, whereas excessive roughness may induce local stress concentration around asperities and increase the tendency toward abrupt post-peak instability. Thus, interface morphology plays a key role in controlling strain localization and the failure response of sandstone–concrete composites.

## 4. Discussion

The results demonstrate that interface roughness plays a dominant role in regulating the mechanical response and failure evolution of sandstone–marble waste powder concrete composites. With increasing *JRC*, both peak stress and elastic modulus increase significantly, indicating that a rougher interface improves interfacial bonding, mechanical interlocking, and load-transfer efficiency. This finding is consistent with previous studies on rock–concrete interfaces, which reported that interface morphology strongly affects stress transfer, crack propagation, and bearing capacity [14,18,19].

From a mechanical perspective, interface roughness affects the failure behavior of the composite through both strengthening and localization effects. At low roughness, the sandstone–concrete interface provides limited geometric constraint, and relative sliding can occur more easily along the interface, resulting in progressive damage accumulation and relatively ductile post-peak behavior. With increasing roughness, the real contact area and asperity interlocking are enhanced, which improves frictional resistance, interfacial bonding, and load-transfer efficiency. However, the asperity geometry also induces local stress concentration near the asperity roots and interface corners. Once tensile cracking, interfacial debonding, and crack coalescence occur in these local regions, the stored elastic energy is rapidly released, leading to abrupt post-peak instability. Therefore, interface roughness has a dual role: it enhances the pre-peak bearing capacity but may also increase the instability of post-peak failure, a finding that is consistent with the energy evolution characteristics observed in this study, where low-roughness specimens exhibit gradual energy dissipation and progressive damage, while high-roughness specimens store more elastic energy before peak stress but experience rapid energy release once interfacial debonding occurs. In the present study, the observed transition from progressive damage accumulation in low-roughness specimens to abrupt post-peak failure in high-roughness specimens similarly reflects this macro-meso coupling, where interface morphology controls both the macroscopic load-transfer efficiency and the mesoscopic crack initiation and coalescence paths, thereby reinforcing the dual role of roughness in enhancing pre-peak strength while increasing post-peak instability.

The AE and DIC results provide complementary evidence for this mechanism. AE responses show that damage evolution changes from continuous accumulation in low-roughness specimens to concentrated release in high-roughness specimens. Meanwhile, DIC strain fields indicate that increasing roughness suppresses interfacial shear slip and promotes tensile-dominated failure; however, excessive roughness may induce local stress concentration around asperities and increase abrupt post-peak instability, although the global SCF decreases. This interpretation is further supported by the quantitative DIC parameters presented in Table 3. The strain concentration factor (SCF) decreases from 3.85 for *J*_1_ to 2.07 for *J*_4_, quantitatively confirming that increasing interface roughness effectively suppresses extreme strain localization. Notably, the *J*_2_ specimen shows the highest e1max (0.143) with a moderate SCF of 2.12, indicating that moderate roughness enhances the tensile deformation capacity while maintaining relatively uniform strain distribution. In contrast, the *J*_1_ specimen exhibits a low e1max (0.102) but a high SCF of 3.85, reflecting that the low-roughness interface cannot effectively transfer load, resulting in highly localized strain concentration and premature interfacial debonding. For *J*_3_ and *J*_4_, the further decreased SCF (2.11 and 2.07) and relatively stable e1max (0.104 and 0.106) suggest that the strain-uniformization benefit of roughness reaches saturation, while excessive mechanical interlocking may induce local stress concentration and abrupt post-peak instability, as also reflected by the AE burst characteristics and energy release behavior. These observations suggest that moderate interface roughness may improve deformation coordination, whereas excessive roughness may increase failure instability.

To further strengthen the sensing-based interpretation, the AE and DIC results were compared from temporal and spatial perspectives. The AE response reflects the temporal evolution of internal damage activity, while the DIC strain field reveals the spatial development of deformation localization. Low-roughness specimens exhibit relatively continuous AE activity and scattered strain localization, indicating progressive damage accumulation. In contrast, high-roughness specimens show concentrated AE bursts near failure and more localized tensile strain bands, suggesting rapid crack coalescence and sudden instability. This AE–DIC consistency indicates that the combined sensing approach can effectively identify the transition from progressive damage to localized unstable failure in sandstone–marble waste powder concrete composites.

Although interface roughness was identified as the dominant factor controlling the mechanical response of the composite, the incorporation of marble waste powder also contributed to the interface behavior. Marble waste powder mainly acted as a micro-filler and nucleation material, improving particle packing density and matrix compactness. A denser concrete matrix can enhance interfacial bonding and reduce local stress concentration around pores, thereby facilitating more effective load transfer across the sandstone–concrete interface. Therefore, the observed improvement in composite performance results from the combined effect of interface roughness and the modified concrete matrix containing marble waste powder.

## 5. Conclusions

(1) Interface roughness significantly improves the mechanical properties of sandstone–concrete composites containing marble waste powder. As the *JRC* increases from 0 to 20.79, the peak stress increases from 18.5 MPa to 50.0 MPa, and the elastic modulus also shows a continuous upward trend. This indicates that higher roughness enhances interfacial bonding stiffness, mechanical interlocking, and load-transfer efficiency.
(2) The energy evolution is strongly affected by interface roughness. At low roughness, damage develops progressively with gradual energy dissipation. With increasing roughness, more elastic energy is stored before peak stress and then rapidly released near failure. Therefore, higher roughness improves the energy storage capacity but also increases the suddenness of post-peak failure.
(3) The AE results show that damage evolution changes from continuous accumulation to concentrated release with increasing roughness. *J*_1_ and *J*_2_ exhibit relatively gradual AE activity, while *J*_3_ and *J*_4_ show sharp increases in AE hits and cumulative AE energy near failure. The RA–AF results further indicate that high-roughness specimens exhibit higher RA values and lower AF values, suggesting increased contributions of frictional sliding and mixed tensile–shear microcracking.
(4) The DIC strain fields indicate that interface roughness controls strain localization and failure mode. *J*_1_ mainly exhibits mixed tensile-shear failure with obvious interfacial slip, whereas *J*_2_–*J*_4_ gradually shift toward tensile-dominated failure. Moderate roughness improves deformation coordination, while excessive roughness may induce local stress concentration and abrupt post-peak instability. Therefore, higher roughness is beneficial for improving the load-transfer capacity of the composite, but its potential effect on post-peak instability should also be considered.

## Figures and Tables

**Table 1:** Stage-based AE response characteristics of specimens with different interface roughness.

Specimen	Low-Activity Stage	Main AE Growth Stage	Burst Stage	Damage Feature
*J*_1_	0–90 s	90–430 s	430–520 s	progressive damage
*J*_2_	20–140 s	140–360 s	360–425 s	progressive-to-localized damage
*J*_3_	80–370 s	370–400 s	400–425 s	rapid crack coalescence
*J*_4_	180–330 s	330–345 s	345–365 s	sudden instability

**Table 2:** Quantitative AE hit statistics and RA-AF parameters of sandstone-marble waste powder concrete composites with different interface roughness.

Specimen	AE Hits	Mean RA/μs·mV^−1^	Median RA/μs·mV^−1^	Mean AF/kHz	Median AF/kHz
*J*_1_	2673	0.1032	0.0211	236.09	78.96
*J*_2_	3527	0.1031	0.0096	238.41	83.33
*J*_3_	1549	0.1731	0.0216	157.77	41.67
*J*_4_	3049	0.1627	0.0122	162.03	47.62

**Table 3:** Quantitative DIC parameters of sandstone-marble waste powder concrete composites at the peak-stress moment.

Specimen	e1max	Surface-Averaged e1	Strain Concentration Factor
*J*_1_	0.102	0.0265	3.85
*J*_2_	0.143	0.0675	2.12
*J*_3_	0.104	0.0493	2.11
*J*_4_	0.106	0.0513	2.07

## Data Availability

The data presented in this study are available from the corresponding author upon reasonable request.

## Abbreviations

The following abbreviations are used in this manuscript:

AE	Acoustic emission
DIC	Digital image correlation
*JRC*	Joint roughness coefficient
ISRM	International Society for Rock Mechanics
